# Awareness and acceptability of gut microbiome transfer

**DOI:** 10.3389/fgstr.2024.1411898

**Published:** 2024-08-09

**Authors:** Ry Yves Tweedie-Cullen, Brooke C. Wilson, José G. B. Derraik, Benjamin B. Albert, Keri Opai, Taygen Edwards, Justin M. O’Sullivan, Wayne S. Cutfield

**Affiliations:** ^1^ Liggins Institute, University of Auckland, Auckland, New Zealand; ^2^ Department of Paediatrics, Child and Youth Health, Faculty of Medical and Health Sciences, University of Auckland, Auckland, New Zealand; ^3^ A Better Start, National Science Challenge, University of Auckland, Auckland, New Zealand; ^4^ Tui Ora, New Plymouth, Taranaki, New Zealand; ^5^ The Maurice Wilkins Centre, University of Auckland, Auckland, New Zealand

**Keywords:** faecal microbiome transfer, public opinion, gastroenterology, survey, microbiome

## Abstract

**Introduction:**

Gut microbiome transfer (GMT or faecal microbiome transfer) is gaining increasing attention as a potential treatment for a range of medical conditions. However, public awareness and acceptance are not well understood.

**Methods:**

To better understand the public perception of microbiome transfer in New Zealand, we undertook a nationwide online survey. The anonymous survey was designed and distributed between 2022-2023. Inclusion criteria included being aged 16 years or older and a resident of New Zealand. Distribution channels included social media advertising, posters in public areas, e-mail newsletters, and a survey facilitation company.

**Results:**

A total of 2441 completed surveys were analyzed. Most respondents (71%) had tertiary education, 59% were female, with 62% identifying as NZ European, 12% as Māori, and 3% as Pacific peoples. The findings identified a high level of awareness and acceptability, with 76% of respondents having heard of GMT, and 96% indicating they would consider it if proven efficacious for a health condition they had. High levels of acceptance were observed across all ethnicities. Encapsulated oral FMT treatment was the preferred transfer method.

**Discussion:**

Primary concerns related to GMT included the diet, health, and screening of stool donors, as well as the demonstration of safety and efficacy. These findings will help inform health professionals and researchers about the public’s needs and preferences regarding GMT.

## Introduction

Faecal microbiome transfer (GMT), also known as faecal microbiota transplant, is a procedure that involves transferring the gut microbiome contained in healthy donor faeces to a recipient with a dysbiotic gut microbiome (1). GMT has been shown to be highly effective in resolving recurrent *Clostridioides difficile* infection (CDI) (2–4) —the most common pathogen responsible for bacteria-induced diarrhoea in hospitalised patients (5). CDI causes marked morbidity and mortality worldwide (6), and GMT can rapidly restore the diversity and functions of the gut microbiome curing clinical disease in these patients (7). GMT is currently the recommended treatment for patients with multiple CDI recurrences (2–4).

GMT has also been trialled as a potential therapy for disorders associated with less severe forms of gut dysbiosis than CDI, such as obesity (8–10), metabolic syndrome (11–14), inflammatory bowel disease (15, 16), irritable bowel syndrome (17), autism (18), and neuropsychiatric conditions (19, 20). Whilst GMT has not been demonstrated to cure these multi-faceted conditions, the resulting alterations in the gut microbiome have been associated with various therapeutic benefits among recipients, including improvements in metabolic syndrome and insulin sensitivity (10–12), intestinal permeability (21), gut inflammation (22), gastrointestinal symptoms (23), and social behaviours (24). As a result, the gut microbiome and GMT have been increasingly covered in the media (25–31), and high-rates of self-administration of GMT have been reported in some patient populations (25, 32).

Patients who received GMT for recurrent CDI have generally reported high levels of satisfaction and indicated it was their preferred treatment for the disease (33). Patients have noted rapid improvements in their symptoms following GMT, and side effects were not only uncommon but also mild and self-limiting (34). However, greater reservation amongst physicians has been noted due to concerns around the potential risks of adverse events, disease transmission, potential adverse alterations of the gut microbiome, the lack of evidence of efficacy, and a belief that patients will be averse to the aesthetics of GMT (34–39). This opinion is widely held amongst clinicians even though there have been few reports of serious GMT-associated adverse events (40). Despite reports of initial aversions to the concept of GMT, surveys to date showed that respondents are interested in learning more (38, 41, 42). Notably, CDI patients provided with efficacy data for their range of possible treatments, usually opted for GMT over other treatments (43). Thus, the concept of using faecal material as a treatment is not a deterrent for recipients (i.e. patients), although the acceptance of GMT depends on the context in which it is offered (38).

Respondents in previous surveys have reported concerns regarding GMT (41, 42, 44, 45), including fears of transmissible infections, the potential financial costs associated with what is portrayed as an experimental therapy, and questions regarding the screening of stool donors’ lifestyle and health status. Amongst specific patient populations, those with ulcerative colitis were reportedly concerned about screening for infections, cleanliness of the GMT procedure, and its efficacy (46, 47). However, when provided with supporting research information and evidence of appropriate donor selection and screening, these patients were willing to consider GMT as a treatment option (44, 48). Further, conditions associated with severe symptoms and lack of effective treatment options (e.g., recurrent CDI) have been shown to be powerful motivators for acceptance of alternative treatments such as GMT (49). However, some patients offered GMT have seen it as a treatment of last resort (41).

Previous surveys on the acceptability of GMT have typically been small or targeted specific populations (33, 36, 50). The largest published survey (n=1828) sampled Chinese medical students (36), and thus may not be broadly representative of wider opinions. Despite strong evidence supporting the efficacy and safety of GMT for CDI (17) and inclusion in treatment guidelines in some countries (3, 4), uptake has been variable (51). In spite of high levels of patient-reported satisfaction following GMT, reservations amongst physicians remains high with close to half of respondents in surveys of physicians expressing concerns, and/or would only considering GMT when traditional methods had been exhausted (34, 52).

Importantly, it is essential to understand the public’s views and potential acceptability of microbiome-related therapies. Identifying ethical, social, and/or cultural issues that may act as barriers or facilitators to GMT acceptability is critical for the design and testing of GMT therapies (42, 45, 46, 50).

## Materials and methods

### Respondent screening and survey design

The survey was designed and administered using an online platform (Qualtrics Labs Inc., Provo, UT, USA). The questionnaire was anonymous, and no personally identifiable information was collected. Potential participants were first screened for the inclusion criteria (age ≥16 years and current residence in New Zealand), and respondents not meeting these criteria were unable to proceed further. Conversely, those meeting the study criteria were then provided with a brief explanation about the study and the type of data being collected (see **Supplementary Table 1**) and given access to a participant information sheet.

The questionnaire comprised 18 questions (see **Supplementary Table 1**), and respondents were not obliged to answer every question. Some questions were associated with a branching logic (specifically Q7/9/11/12/13/17/18), so that certain questions were displayed or not depending on the respondent’s answer. Multiple choice or ranked questions (e.g. Q14) were presented to each respondent in a random order to each respondent to avoid presentation bias.

Demographic data recorded included the participant’s gender, age range, highest completed qualification, and ethnicity. The latter was self-reported, and the respective question allowed respondents to choose multiple ethnicities. However, respondents were allocated to a single prioritised ethnicity using the NZ Ministry of Health hierarchical system of classification (53) as follows: Māori, Pacific Peoples, European, Chinese, Indian, Other Asian, “MEELA” (Middle Eastern, Latin American, or African) and New Zealand European.

### Survey distribution

The survey was carried out over 5 months (September 2022 until January 2023) with a link distributed through a range of channels. These included targeted advertisements (based on geography, age, or gender) on Facebook and Instagram, e-mail newsletters sent out within the University of Auckland, posters displayed in public areas throughout New Zealand, and through distribution of physical flyers to letterboxes. To ensure we had a more balanced ethnic, gender and educational background of respondents, an additional 200 paid responses were provided by a survey facilitation company (Dynata LLC, New Zealand branch).

### Quantitative analyses

Only data on submitted surveys underwent quantitative analyses. Data were analysed using SAS (v9.4, SAS Institute Inc., Cary, NC, USA) and Prism (v.9.5.1, GraphPad Software, San Diego, California USA). All tests were two-tailed and statistical significance set at p<0.05). “The likelihood of a binary outcome (e.g., answering “Yes” to a given question) was examined using multivariable generalized linear regression models based on a Poisson distribution, with effect sizes expressed as the adjusted relative risks (aRR) and respective 95% confidence intervals (CI). Models adjusted for the participant’s age band and level of education (both as ranked continuous variables), as well as gut issues and IBD (both coded as either “yes” or “no” for each respondent).

### Thematic analyses

Five questions allowed respondents to provide free text responses (see **Supplementary Table 1**): Q11, Q12, Q13, Q17 and Q18. Reflexive thematic analyses (54) were performed on these free text responses using NVivo (v20.7.1, QSR International Pty Ltd., MA, USA). Q13 was excluded for having only three responses recorded.

### Ethics

Ethics approval was granted by the University of Auckland Human Participants Ethics Committee (UAHPEC24594). Informed consent was only deemed as provided when respondents began the survey.

## Results

### Survey respondents

A total of 2441 submitted surveys were analysed. Responses were received from a diverse range of respondents in terms of gender, age, ethnicity, and highest educational level achieved (see **Table 1**). The majority of respondents (63%) rated their health as good to excellent, and 8% as poor or very poor. Most respondents who had underlying medical conditions self-rated these as mild or moderate (see **Supplementary Figure 1**).

**Table 1:** Demographic characteristics of 2441 respondents of a nationwide online survey on GMT knowledge and acceptance.

Demographic Characteristic	Sub-category	Frequency (n) (Percentage (%))
**Ethnicity^1^**	New Zealand European | Pākehā	1524 (62.4%)
	Māori	292 (12%)
	European	166 (7.6%)
	Pacific Peoples	83 (3.4%)
	Chinese	71 (2.9%)
	Other Asian	67 (2.7%)
	Indian	56 (2.3%)
	MELAA	51 (2.1%)
	Prefer not to say	27 (1.1%)
	Not stated/No Response	86 (3.5%)
**Gender**	Female	1442 (59.1%)
	Male	858 (35.1%)
	Other	37 (1.5%)
	Prefer not to say	17 (0.7%)
	Not stated	87 (3.6%)
**Age**	16–25 years	245 (10.0%)
	26–35 years	525 (21.5%)
	36–45 years	432 (17.7%)
	46–55 years	446 (18.3%)
	56–65 years	418 (17.1%)
	≥66 years	294 (12%)
	Not stated	80 (3.4%)
**Education^2^**	Post-graduate diploma or degree	1090 (44.7%)
	Bachelor (undergraduate) degree	641 (26.3%)
	Trade / technical / vocational	290 (11.9%)
	High school	278 (11.4%)
	Less than high school	55 (2.3%)
	Not stated	86 (3.6%)

Data are *n* (%). MELAA, Middle Eastern, Latin American, or African.

^1^Self-reported ethnicity according to a hierarchical system of classification.

^2^Highest completed qualification.

### Awareness of GMT

**Figure 1:**
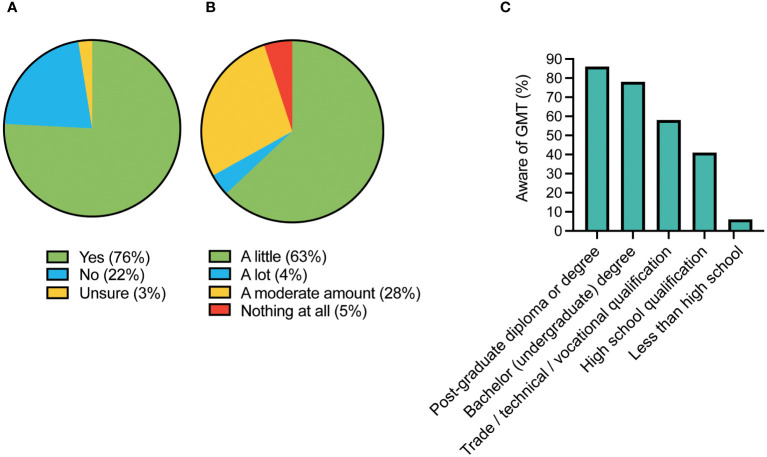
Awareness of GMT amongst survey respondents. **(A)** Overall awareness of GMT; **(B)** Knowledge of GMT amongst those reporting awareness of it; **(C)** GMT awareness in association with the respondent's highest level of education.

Awareness of GMT was high with 76% of respondents having heard of GMT previously (see **Figure 1A**). Respondents in this group also reported high knowledge with only 5% knowing “nothing at all”, and 32% “knowing a lot” or “a moderate amount”. Higher education level and greater age were associated with awareness of GMT (p<0.001 for both). Of those with a bachelor’s or post-graduate degree, 83% were aware of GMT versus only 34% of those without (see **Figures 1A–C**).

### Willingness to undergo GMT

**Figure 2:**
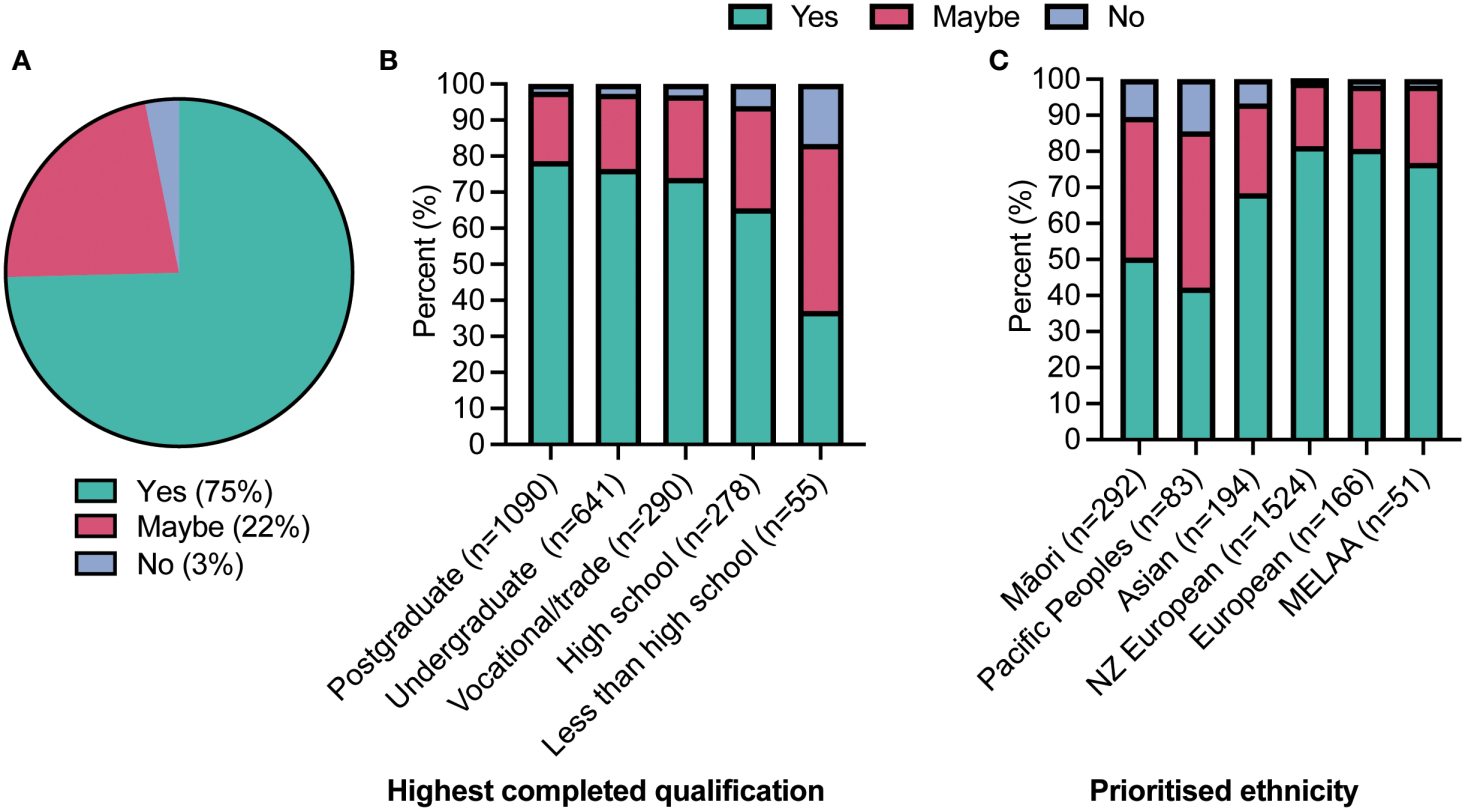
The willingness of respondents to undergo faecal microbiome transfer (GMT). **(A)** If GMT was proven effective for a condition they had; **(B)** According to their highest level of education (p<0.0001 from a Chi-square test); and **(C)** according to their prioritised ethnicity (p<0.0001).

After being presented with a brief overview of GMT, respondents were asked if they would consider undergoing the procedure if proven effective for a health condition affecting them. Almost all (95%) were receptive to undergoing GMT if it could effectively treat their condition, with 73% and 22% responding “yes” or “maybe”, respectively (**Figure 2A**). Although receptiveness to GMT was high overall, there was greater willingness towards GMT amongst the highly educated respondents (p<0.001; **Figure 2B**). No association was observed between overall reported health status or illness severity and willingness to undergo an GMT (see **Supplementary Figure 1**) (p=0.32). However, those reporting gut issues were more willing to undergo GMT than those without (p<0.001).

Acceptability amongst Māori (indigenous peoples) was similarly high, with 90% being receptive (“yes” or “maybe”) to GMT if it was appropriate. A similar pattern was seen amongst Pacific Peoples, with 85% being open to GMT. However, slightly more respondents from both these groups were more likely to select “no” when asked whether they would undergo an GMT, compared to other ethnic groups surveyed (p<0.001) (see **Figure 2C**). Importantly this difference remained when taking into account education levels, age, and the presence of gut issues (p<0.05). When queried on “Cultural or religious values that would affect your decision?” only three responses were received which mentioned spiritual beliefs.

**Figure 3:**
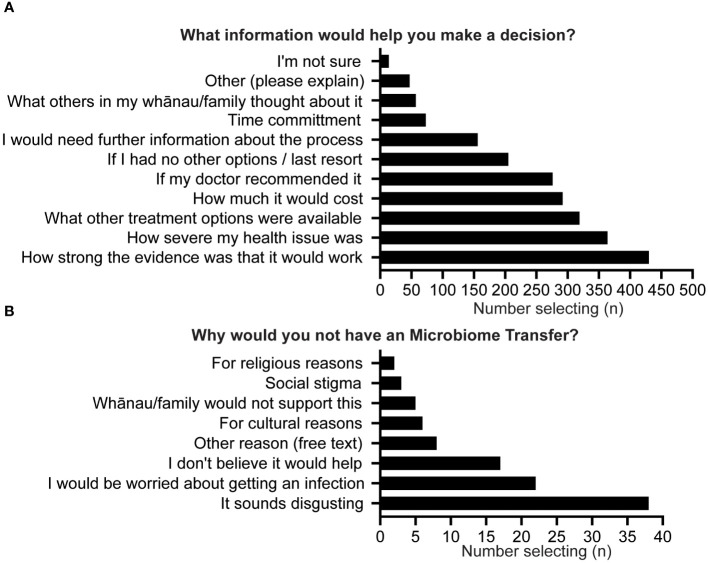
**(A)** What information would help you decide; **(B)** Why would you not have an GMT.

Those respondents who would “maybe” consider undergoing GMT (22%) were asked a follow-up question regarding what information would help their decision-making process. The three most reported responses to this were the strength of the evidence of GMT effectiveness (81%), the severity of their health issue (69%), and the other treatment options available (60%) (see **Figure 3**). Notably, social/cultural acceptance (i.e., “What others in my whānau/family thought about it”) was only chosen by 11% of respondents. A qualitative analysis of the 47 free text answers to this question demonstrated themes related to worries about potential side effects, safety, and efficacy. They also showed concerns regarding the transfer of undiagnosed conditions and overcoming discomfort with ingesting faecal matter. Safety concerns encompassed the presence of “unhealthy bacteria” and the donor’s health. Respondents favoured capsules for delivery and emphasised understanding the source and processing of donor stool.

The 3% of respondents who would not undergo GMT were asked a follow-up question about their reasoning. Half selected “It sounds disgusting” as a justification; 29% reported concerns about the risk of infection, while 23% of respondents did not believe GMT would be effective (see **Figure 3B**). A qualitative analysis of 14 free text responses to this question covered themes related to personal dietary preferences, with some individuals hesitant due to donor diet. Others cited the personal absence of any health conditions, an aversion or discomfort with the concept, and a lack of familiarity with GMT. Scepticism about the effectiveness of microbiome transfers for chronic illnesses was mentioned, as was a preference for natural approaches to microbiome health. Safety and potential side effects were also raised as concerns, with respondents indicating a need for proof of safety before considering such a procedure.

### Administration routes

**Figure 4:**
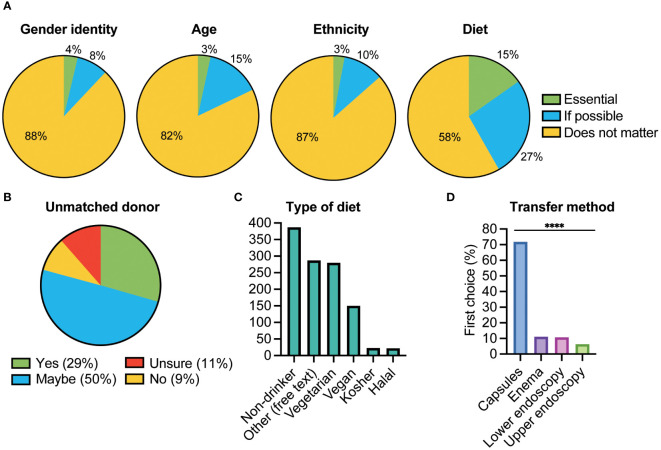
Donor preferences and administration route. **(A)** Respondents were asked whether it was “essential”, “if possible” or did not matter” regarding matching themselves with a potential stool donor in terms of Gender identity, Age, Ethnicity, or Diet; **(B)** Whether they would still accept an GMT from an unmatched donor; **(C)** What preferences they had in terms of their donor’s diet. Other refers to free text responses that respondents could enter; **(D)** Respondents were asked to rank the four alternatives 1-4, the percentage ranking each option as their first choice is shown. There was a significant difference between the four options with GMT being delivered via capsule the preferred option (****p <0.0001).

When survey respondents ranked their preferred method of GMT, a significant majority ranked capsules as their first choice (72%). In contrast, only 11% preferred enema, 11% lower endoscopy, and 6% upper endoscopy (p<0.0001) (see **Figure 4D**).

### Donor preferences

Amongst respondents willing to undergo GMT, the primary concern was that donors would be “healthy and appropriate” (71%). Approximately 1 in 4 (27%) would prefer anonymous donors, but only 6% preferred donors they knew. Respondents were also asked about the importance of having stool donors ‘matching’ their preferences on “gender identity”, “age”, “ethnicity,” or “diet”. A significant proportion (p<0.001) of respondents expressed that it was either “essential” (15%) or “if possible” (27%) to be matched based on diet (see **Figure 4A**). Only 3% of respondents indicated that it was essential to be matched on the other characteristics (ethnicity 3%, age 3%, gender 4%). Primary dietary preferences included being a non-alcoholic drinker and having a vegetarian or vegan diet (280 and 150 respondents respectively) (see **Figure 4C**). A total of 281 free text answers to this question were recorded. A qualitative thematic analysis of respondents’ preferences for the donor’s diet revealed a variety of expectations. Themes included a desire for a healthy diet, emphasising factors like vegetables, low fat, low sugar, and whole foods. Respondents mentioned avoiding processed foods, alcohol, smoking, and illicit drugs as important criteria. Some emphasised the importance of dietary diversity, particularly a plant-based diet akin to the Mediterranean diet, for enhancing microbiome richness and resilience. Others indicated a preference for a donor with a diet similar or “compatible” to their own, citing personal taste and medical conditions, such as coeliac disease or food allergies.

Among the 462 respondents reporting at least one “essential” preference regarding stool donor characteristics, 79% remained open to undergoing GMT even if donors didn’t match their preferences. Only 9% stated that they would refuse an GMT unless they could be matched with a donor who met their preference (see **Figure 4B**). A total of 10 respondents gave free text answers as to this question, a qualitative analysis demonstrated that respondents prioritised medical advice, treatment effectiveness, personal preferences (such diet and lifestyle), and drew parallels to blood transfusions.

## Discussion

This large nationwide survey of adults in New Zealand found a high level of awareness of GMT and a striking level of acceptability and willingness to undergo GMT if it could be of clinical value. Of those unwilling to undergo GMT, some of their concerns could likely be addressed, albeit technically challenging, by donor-recipient diet matching. Respondents raised the importance of support from their physician, and the need for evidence of safety and clinical efficacy in considering its use for their medical condition/s. Whilst most respondents had minimal expectations of donors, some specified the need for considerations such as diet and health. The high level of awareness seen in our survey can likely be attributed to the increasing attention beyond medical literature (e.g. online and in traditional media) directed at the more health literate (27–29, 39) and recent surveys have also shown a rise in awareness (39, 55).

Cultural and religious aspects of GMT have been examined in previous studies (44, 56), identifying concerns in terms of informed consent, specifically regarding recipients being aware of the donor’s diet, alcohol intake, and religion (56). Our study indicated that Māori showed were more likely to decline or express uncertainty about GMT compared to most ethnic groups, excluding Pacific Peoples. This reluctance may be attributed to historical and cultural factors, institutional bias and discrimination, socioeconomic disparities, health literacy, and a lack of cultural safety and representation in healthcare (57). These factors may ultimately lead to mistrust, limited access, and inadequate culturally appropriate care for Māori. Addressing these issues requires comprehensive approaches that prioritise cultural safety, equitable access, and inclusion of Māori voices in healthcare decision-making.

The survey indicated that most respondents preferred oral encapsulated GMT compared to other delivery methods. Previous studies have primarily focused on nasogastric, upper/lower endoscopy, or enema (44, 47, 49, 50), but when given the choice, patients generally prefer capsules, perceiving it to be more acceptable and less unpleasant (43, 46, 55, 58). For instance, a higher proportion of patients receiving GMT via capsules reported their experience as “not at all unpleasant” compared to those undergoing colonoscopy (59). Encapsulated GMT offers additional benefits such as being non-invasive, easier to deliver, carrying a lower risk of procedure-related complications, and not requiring sedation or anaesthesia. Meta-analyses have shown that encapsulated GMT is comparable to other methods in terms of clinical outcomes for CDI (17, 60). Moreover, its ability to be self-administered in a clinic or at home enhances convenience and accessibility.

At present GMT is performed using stool from a human donor (15). The donor health status (including colonisation by pathogens) is important when considering GMT, necessitating stringent testing of any potential donor (3, 15, 61–64). Patient views on the selection of stool donors have been explored in previous research and demonstrated that major concerns from potential recipients relate to donor selection and screening (44, 45, 52, 65). In our study, respondents emphasised having knowledge of the source of faecal matter, what donor health checks were undertaken, and assurance regarding the possible transfer of harmful pathogens. Most preferred a donor with a healthy, vegetable-rich diet, limited alcohol intake, and non-smoking habits, similar to perspectives reported previously (56). Although many were indifferent to the diet of a healthy donor, some preferred donors with diets similar to their own, such as vegetarian or vegan (see **Figure 4C**). The responses underscore an interest in the donor’s health, diet and facilitation of diet matching was indicated as essential for a small number of respondents (see **Figure 4B**). In agreement with previous research, this survey demonstrated that many people compare GMT to a blood transfusion (44, 49) supporting this being an generally acceptable approach in the future (66).

We additionally wanted to explore whether involvement in donor selection would be a facilitator or barrier to the adoption of GMT and what other selection criteria were important. Most respondents would prefer a “healthy and appropriate donor” and/or a preference for an anonymous donor, with a smaller proportion (6%) preferring a donor whom they knew. This is in line with previous studies, for instance, a survey of 183 GMT-naïve patients reported that 28% of patients found the prospect of needing to select their own donor as too unappealing to consider GMT as a treatment with all respondents preferring an unrelated anonymous donor (43). Other studies have also demonstrated that patients preferred their doctor to decide on the appropriate stool donor/s (47, 55). Conversely, some studies have also reported that many respondents (38-80%) would prefer to have a family member/spouse as their donor (45, 47, 49).

Both higher levels of education and older age were associated with greater awareness and willingness to undergo GMT amongst our respondents, consistent with previous research (33, 43). Education has previously been demonstrated as a key determinant of health literacy and engagement (67). The high proportion of respondents with a vocational or higher education (83%) could indicate a greater familiarity with the science behind GMT, leading to a better understanding of its potential benefits and risks. In addition, higher levels of education have been shown to increases the likelihood of trusting medical professionals and research (67). Older individuals may also be more accepting due to experience with chronic gastrointestinal disorders. Further, studies have also demonstrated an association between education levels and knowledge about and utilisation of complementary and alternative medicine (44, 68). Many survey respondents associated GMT as a “natural” therapy, which made them more receptive to it. This perception of GMT as a natural treatment aligns with previous observations comparing GMT and probiotics, which were also considered attractive due to their perceived natural or holistic nature (38, 41, 42, 44). Conversely, younger individuals may be less accepting of GMT due to limited exposure, fewer chronic issues, and perceived social stigma relating to its unconventional nature (41).

In our survey we saw no association between overall self-reported health status and willingness to undergo GMT (see **Supplementary Figure 1**). However, those respondents who reported having “gut issues” were more willing to undergo an GMT than those without. The perceived severity of the health condition that could be treated by the GMT has previously been shown to increase acceptance (44). For instance, 89% of people with well-controlled ulcerative colitis (85/95) were either willing or would maybe undergo GMT, with only 11% being unwilling, in addition previous hospitalisation for their condition was linked to increased willingness (47). Further, whilst initial distaste has frequently been reported as a barrier to GMT, disease burden, perceived benefits, and desire for an effective treatment have all been shown to be greater motivating factors (33, 44). Patients with CDI had higher willingness to undergo GMT than healthy controls, with greater willingness in those with multiple episodes of CDI (69). In addition, patients in previous surveys with chronic gastrointestinal disorders were likely more willing to try GMT if they perceived their current treatments as ineffective or unsatisfactory (41).

Respondents in our study emphasised the need to understand the safety, efficacy, and potential complications of GMT. Amongst those unwilling to undergo an GMT, the primary reasons were due to concerns about ingesting faecal matter or lack of perceived benefit. Similarly, research has shown that a large portion of physicians are reluctant to recommend GMT (4, 34, 52) due to concerns about long-term safety (38, 70, 71), the potential impacts of altering the gut microbiome (26, 28–31), and lack of clinical utility beyond CDI. For our respondents, the doctor-patient relationship and medical advice were crucial for acceptance of GMT, aligning with previous research (44). Whilst many prioritised medical advice, personal preferences and specific medical conditions also influenced their decisions, in line with findings from earlier studies (41, 43).

There are several limitations associated with this study. Firstly, the respondents in the study were primarily individuals with a higher level of education. However, the willingness to participate in the study was relatively similar across different education levels beyond high school/college. Furthermore, the study was conducted in New Zealand, which may affect the generalisability of the findings to an international context. Nevertheless, most respondents were of European descent (New Zealand European/Pākehā), increasing the relevance of the results to other Western countries with similar demographic profiles. It should be noted that although the respondents did not fully represent the ethnic diversity of New Zealand, the sample size was adequate to detect any potential ethnic differences in willingness to undergo GMT. Notably, this study is one of the largest conducted on this topic to date and additionally, it diverged from prior research that has targeted specific subgroups, such as patients with chronic gut issues or medical students, by instead surveying the general public.

This survey gives new insights into the views and attitudes of the public in New Zealand and has implications for the possible future development and application of GMT as a therapeutic modality if utility beyond CDI can be demonstrated. Whilst the survey demonstrated both high awareness and willingness regarding GMT, this contrasts with greater reservations amongst physician and demonstrates a need for greater education, research, and communication efforts to better inform clinicians and the public about GMT to address concerns and misconceptions. It also indicates that there may be segments of the population for which the design and delivery of personalised and patient centred GMT interventions are warranted. Ultimately, greater availability of GMT is dependent not only on institutional acceptance and provision but also demonstration of clinical utility and efficacy.

## Data Availability

Anonymous respondent response data will be made available at date of publication upon valid requests to the Liggins Institute’s Data Oversight Committee. Requestors will need to provide a methodologically sound proposal, obtain appropriate ethical approval, and sign a Data Access Agreement. The data access agreement will include a commitment to using the data only for the specified proposal, not to attempt to identify any individual respondent, a commitment to secure storage and use of data, and to destroy or return the data after completion of the project.

## Supplementary material

Supplementary Figure 1
**(A)** Self-reported and self-scored condition severity. Each column represents the individuals who reported they had a specific condition and their proportional severity rating; **(B)** Overall self-rated health; **(C)** There was no significant difference regarding willingness of people to undergo GMT who either reported no health conditions, or rated at least one severe, moderate, or mild; **(D)** Willingness to undergo an GMT and overall self-reported health, ns.
