# Supplementary figures and images for: Awareness and acceptability of gut microbiome transfer
Source: Front Gastroenterol (Lausanne). 2024 Aug 9;3:1411898. doi: 10.3389/fgstr.2024.1411898 (PMC12952461; doi:10.3389/fgstr.2024.1411898)

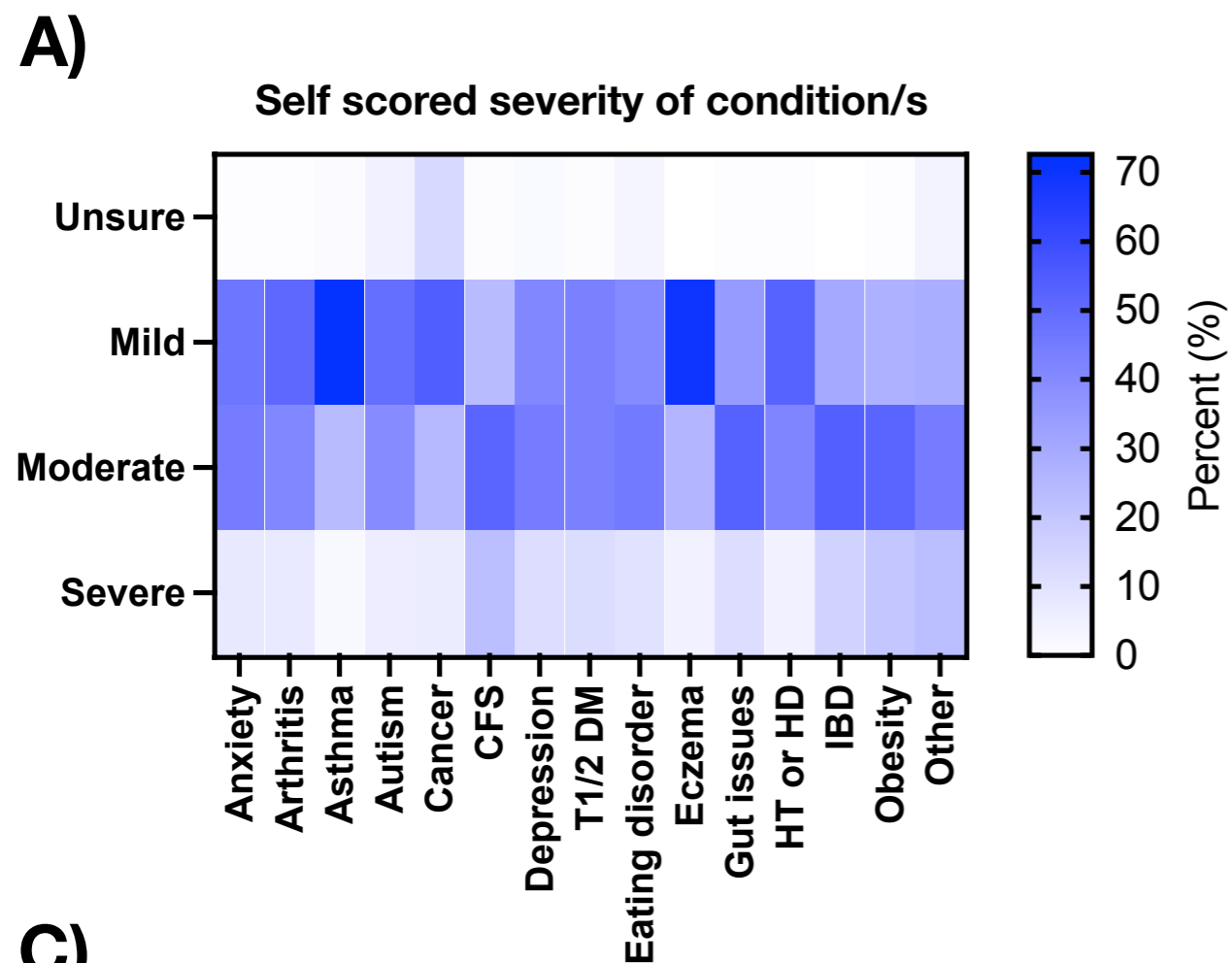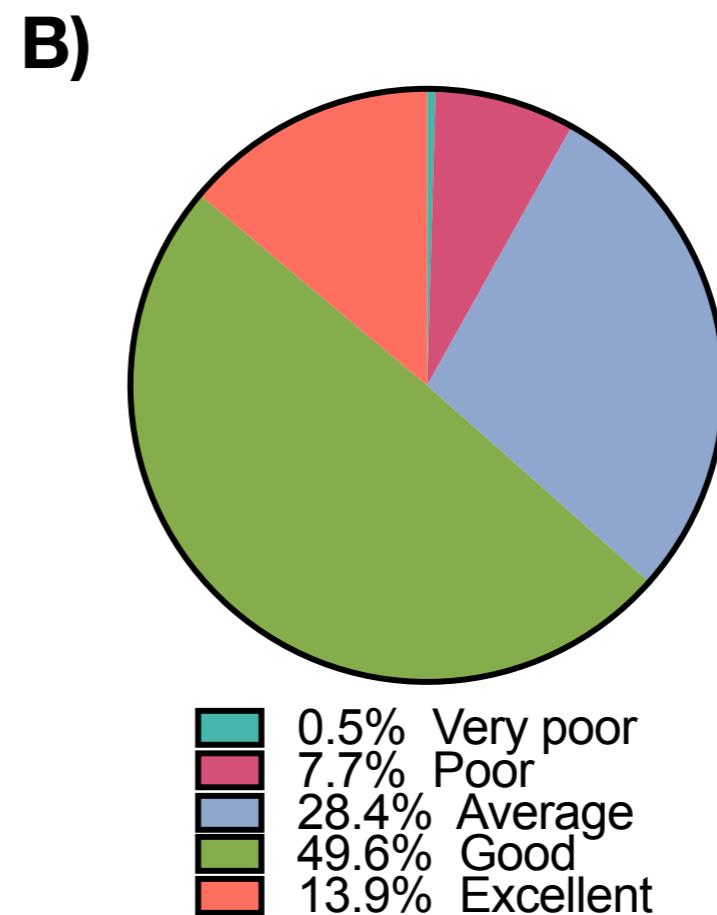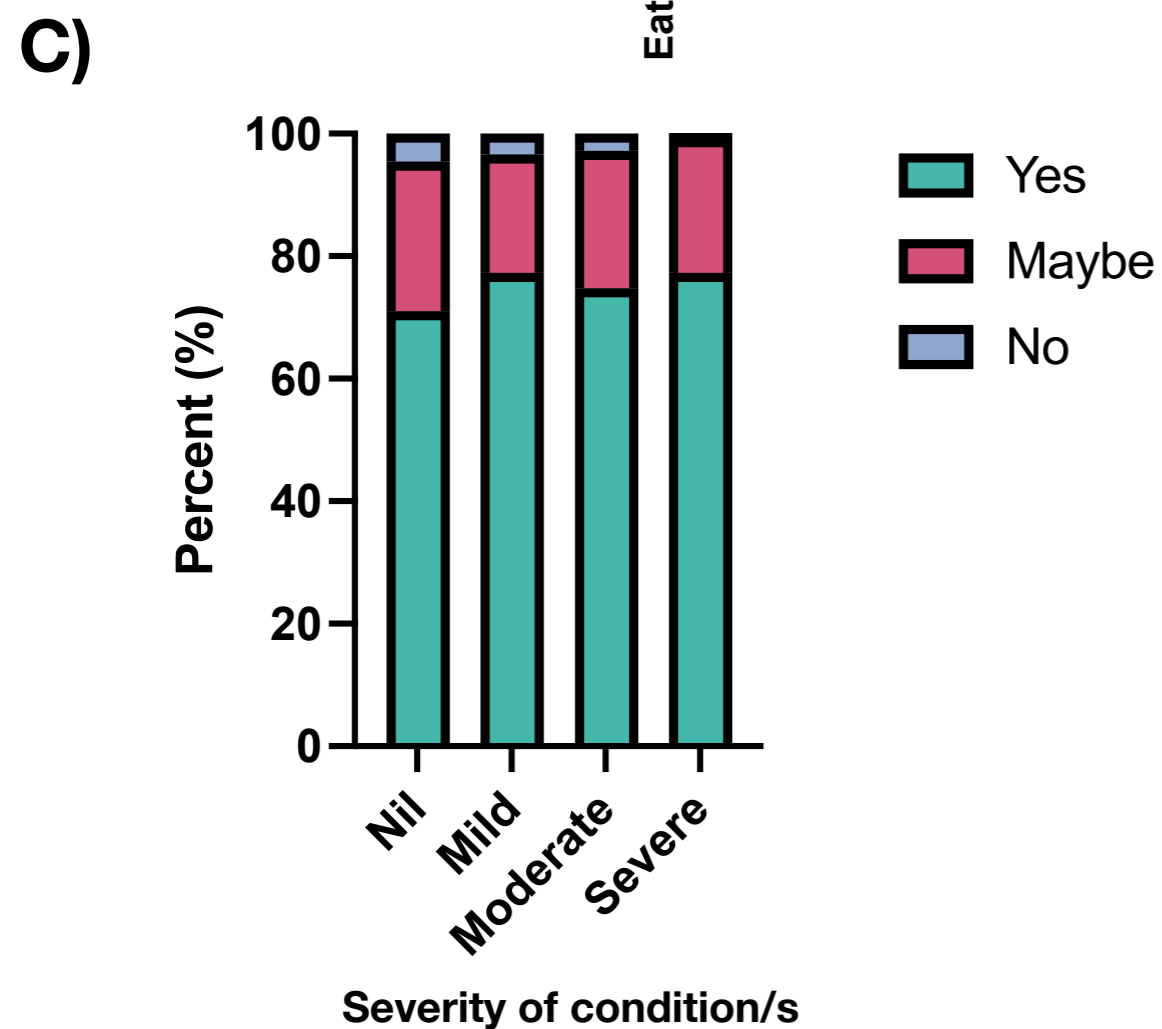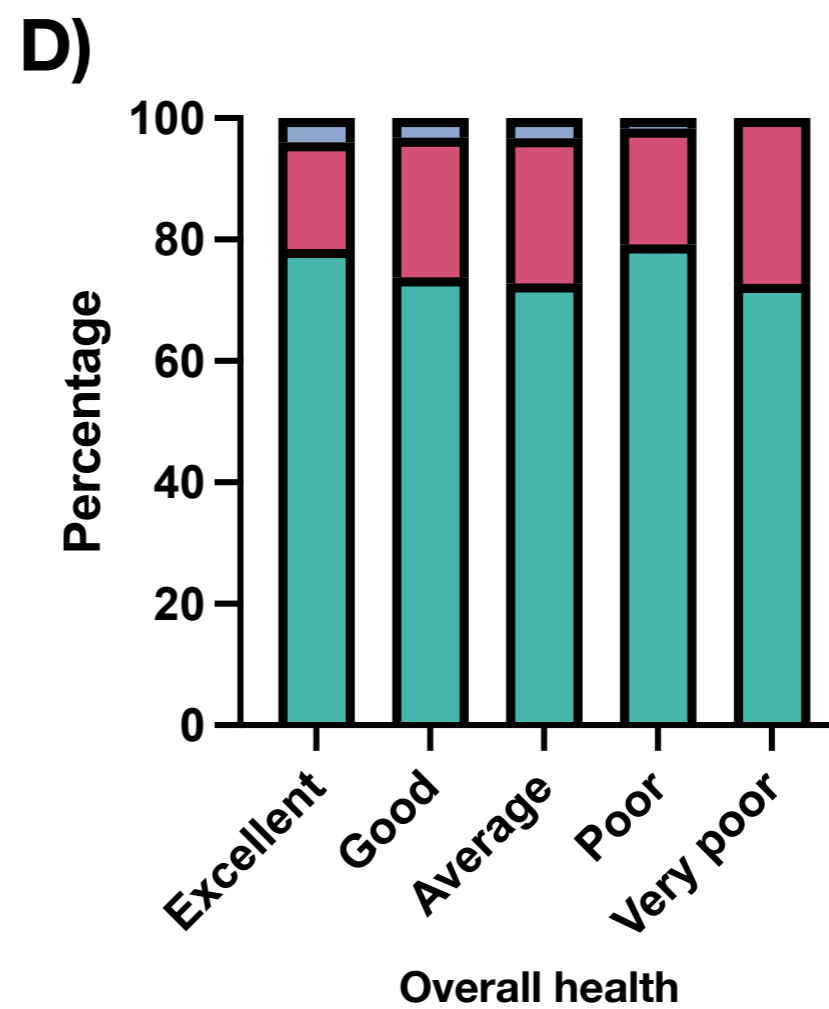

Supplement: Supplementary Figure 1 — (A) Self-reported and self-scored condition severity. Each column represents the individuals who reported they had a specific condition and their proportional severity rating; (B) Overall self-rated health; (C) There was no significant difference regarding willingness of people to undergo GMT who either reported no health conditions, or rated at least one severe, moderate, or mild; (D) Willingness to undergo an GMT and overall self-reported health, ns. [file DataSheet_1.pdf]
