# Supplementary material for: Awareness and acceptability of gut microbiome transfer
Source: Front Gastroenterol (Lausanne). 2024 Aug 9;3:1411898. doi: 10.3389/fgstr.2024.1411898 (PMC12952461; doi:10.3389/fgstr.2024.1411898)
Supplement: Supplementary file 2 [file Table_1.docx]

**Supplementary Table 1: Outline of the survey utilised in this study.**

| **Screening Questions** |  |
| --- | --- |
| **We are currently only collecting the views of adults (16 years or older) who live in Aotearoa / New Zealand.**   **Are you over the age of 16 and currently live in Aotearoa New Zealand?** | **Yes**  **No** |
| **Introduction Section** |  |
| **Gut Microbiome Transfer Survey**  Tēnā koutou katoa  Worldwide, many clinical trials are studying**gut microbiome transfers**(sometimes referred to as 'faecal transplant') as a treatment for common health conditions such as diabetes, obesity, eczema, asthma and gut and eating disorders.  We want to understand people's views in Aotearoa / New Zealand on microbiome transfers: how you feel about it, and whether you think it would be an acceptable medical treatment for certain health conditions.  We will ask you questions about: • Aspects of your health which may be affected by the gut microbiome • What you know and think about microbiome transfers • Your demographics   Your views will be valuable in helping develop this new form of medical treatment.  Click to download the Patient Information Sheet (PIS).  You will only have consented to join the study when you click the ‘submit’ box at the end of the survey.  Ethics approval has been granted by the University of Auckland Human Respondents Ethics Committee in August 2022 for a period of 3 years (approval number UAHPEC24594). | |
| **Q1) Gender** |  |
| **What is your gender identity?**(select all that apply) | **Male / Tāne**  **Female / Wahine**  **Another gender (please specify)**  **Prefer not to say** |
| **Q2) Age** |  |
| What is your age range? (select one) | **16 - 25 years**  **26 - 35 years**  **36 - 45 years**  **46 - 55 years**  **56 - 65 years**  **66+ years** |
| **Q3) What ethnicity do you identify as?**(select all that apply) | **Māori**  **New Zealand European / Pākehā**  **Pacific Peoples**  **Indian**  **Chinese**  **Other Asian**  **Middle Eastern / Latin American / African**  **Other (please specify)**  **Prefer not to say** |
| **Q4) Education** |  |
| **What is your highest completed education qualification?** (select one) | **Post-graduate diploma or degree**  **Bachelor (undergraduate) degree**  **Trade / technical / vocational qualification**  **High school qualification**  **Less than high school** |
| Health Section |  |
| **The following questions ask you about aspects of your health which are potentially affected by the gut microbiome.** |  |
| **Q5) How would you describe your overall health and wellbeing?** (select one) | **Excellent**  **Good**  **Average**  **Poor**  **Very poor** |
| **Q6) Do you have any of the following health conditions?**(select all that apply) | **Anxiety**  **Arthritis**  **Asthma**  **Autism (ASD)**  **Cancer**  **Chronic Fatigue Syndrome**  **Depression**  **Diabetes (any type)**  **Eating disorder (e.g. anorexia, bulimia)**  **Eczema**  **Gut issues (e.g. irritable bowel syndrome, frequent abdominal pain, constipation, diarrhoea)**  **Hypertension or heart disease**  **Inflammatory bowel disease (e.g. Crohn's disease, ulcerative colitis)**  **Obesity**  **Other (please describe)** |
| **Q7) Please rate the current severity of your health condition/s**  ***(Displayed if any conditions selected)*** | **Mild**  **Moderate**  **Severe**  **Unsure** |
| **Q8) Have you ever heard of a "microbiome transfer" before?** This is sometimes referred to as "faecal transplant". (select one) | **Yes**  **No**  **Unsure** |
| **Q9) How much do you know about microbiome transfers?** (select one)  ***(Displayed if “Yes” selected in previous question)*** | **Nothing at all**  **A little**  **A moderate amount**  **A lot** |
| **Please read this explanation of microbiome transfers. We will then ask you about your thoughts regarding it.**  The **gut microbiome** is the community of microbes, such as bacteria, that live in our gut. These microbes help us digest food, make vitamins, and can regulate appetite & mood. They are thought to play a role in obesity, diabetes, bowel disease, eczema, asthma & eating disorders. Keeping our gut microbiome healthy may help to keep us healthier.  A **microbiome transfer** is the process by which gut microbes are moved from one person to another. The easiest way of isolating the gut microbiome is by using stool (poo). A healthy donor is first screened to ensure they have no infections or medical conditions, and gut bacteria isolated from their stool. The bacteria is then transferred in several ways. Either as capsules which are swallowed, or using a flexible tube that goes down your throat or placed in the back passage.  Microbiome transfers are an effective treatment for severe diarrhoea, working better than antibiotics. It is also being studied as a treatment for various other health conditions. It is important to know what you think about the topic to guide development of it as a treatment. |  |
| **Q10) Now that you have read some information on microbiome transfers, would you consider having a microbiome transfer if it was proven effective for a health condition you had?** (select one) | **Yes, definitely**  **Maybe / I don't know (it depends...)**  **No, definitely not** |
| **Q11) What information would help you make a decision?** (select all that apply)  ***(Displayed if “Maybe / I don’t know” selected in previous question)*** | ***(Display order randomised)***  **I would need further information about the process**  **How severe my health issue was**  **How strong the evidence was that it would work**  **What others in my whānau/family thought about it**  **What other treatment options were available**  **How much it would cost**  **Time commitment**  **If I had no other options / last resort**  **If my doctor recommended it**  **I'm not sure**  **Other (please explain)** |
| **Q12) Why would you not consider having a microbiome transfer?** (select all that apply)  ***(Displayed if “No” selected in Q10)*** | ***(Display order randomised)***  **It sounds disgusting**  **I don't believe it would help**  **I would be worried about getting an infection**  **My whānau/family would not support this**  **For cultural reasons**  **For religious reasons**  **Social stigma**  **I'm not sure**  **Other reason (please explain)** |
| **Q13) We would appreciate knowing more about your cultural or religious values that would affect your decision?** (optional)  ***(Displayed if “For cultural reasons” or “For religious reasons” selected in previous question)*** | **freetext** |
| **Questions about microbiome transfer preferences:**  **We are now going to ask you some questions about your preferences if you were going to have a microbiome transfer.** |  |
| **Q14) Which transfer method would you prefer?**Please drag to arrange from most preferable (1) to least preferable (4). | ***(Display order randomised)***  **Capsules**  **(swallowing approximately 30 capsules, split over 2 days, in a clinic)**  **Enema**  **(through a tube up the back passage, performed in a clinic but not under sedation)**  **Lower endoscopy**  **(through a tube up the back passage and into the gut, while in a clinic under sedation)**  **Upper endoscopy**  **(through a tube down the throat and into the gut, while in a clinic under sedation)** |
| **Q15) Would you prefer the donor to be...** (select all that apply) | ***Anonymous***  ***Someone I know (e.g. a whānau/family member, partner, friend)***  ***Don't mind, as long as the donor is healthy and appropriate***  ***Unsure*** |
| **Q16) Would it be essential that you were matched with your donor based on...** | ***Essential If possible Does not matter***  ***Age***  ***Diet***  ***Ethnicity***  ***Gender identity***  ***Other factor/s (please specify)*** |
| **Q17) What sort of diet would you want your donor to have?**(select all that apply) | ***Vegetarian***  ***Vegan***  ***Kosher***  ***Halal***  ***Non-drinker (alcohol)***  ***Other (please specify)*** |
| **Q18) If the most effective donor did not match any of your preferences would you still accept the treatment?**(select one) | ***Maybe***  ***No***  ***Unsure (free text)*** |
